# From process to publication: the conduct and reporting of co-design in health research in the Western Pacific region

**DOI:** 10.1016/j.lanwpc.2025.101766

**Published:** 2025-12-05

**Authors:** Yaqoot Fatima, Michelle Olaithe, Shannon Edmed, Bushra Nasir

**Affiliations:** a Thompson Institute, University of the Sunshine Coast, Sunshine Coast, Queensland, Australia; b Child Health Research Centre, The University of Queensland, Brisbane, Australia; c School of Psychological Science, University of Western Australia, Western Australia; d ARC Centre of Excellence for Children and Families Over the Life Course, The University of Queensland, Brisbane, Australia; e Medical School, Faculty of Health, Medicine and Behavioural Sciences, The University of Queensland, Toowoomba, Queensland, Australia

In recent years, health research in the Western Pacific region has seen a rise in the use of co-design as a research approach.^1^ While the growing acceptance and use of co-design research is encouraging, inconsistencies in how co-design is defined, implemented, evaluated, and reported often make it difficult to determine the extent to which co-design principles are genuinely applied.^2^ Addressing this variability is essential for ensuring transparency, comparability, and robust evaluation of co-designed health interventions.

As a transformative method rooted in consumer leadership, co-design is critical for consumer-centred and culturally grounded health research.^3^ While terms such as “co-creation”, “co-production”, and “co-design” are often used interchangeably, co-design specifically emphasises collaborative problem-definition and solution-development between researchers and end-users across all project stages, whereas co-production tends to focus on shared implementation and delivery.^2^ This distinction is crucial for ensuring that the principles of genuine co-design, particularly consumer leadership and co-creation of knowledge, are not diluted in practice. This is especially important when working with Indigenous communities, where the historical and ongoing effects of colonisation and the dominance of Western biomedical methods restrict authentic research with and for Indigenous peoples.^4^

Conventional “participatory” or “consumer-informed” approaches often fail to capture the depth of relational, cultural, spiritual, and contextual factors critical for the success of co-design initiatives. For genuine co-design, the process must go beyond merely involving consumers as passive participants. Embedding end-user leadership in defining research priorities, shaping methods, co-creating solutions, and endorsing outputs is vital to ensure that the co-design results are responsive to consumers’ needs and priorities.^5^ The key to fostering consumer leadership is ongoing dialogue, shared decision-making, and a commitment to long-term engagement, often starting well before the project begins and continuing beyond completion.^6^

In Indigenous communities, genuine co-design requires a relational process prioritising transparency, respect, mutual benefit, and applying research methods rooted in Indigenous ways of knowing, being, and doing.^7^ This includes strengths-based and culturally grounded approaches that encourage critical reflexivity, create inclusive spaces where Indigenous and Western knowledge systems can meet respectfully.^8^ These methods privilege communities lived experiences, histories, and epistemologies while upholding scientific rigour and ethical integrity.

Strong emerging evidence shows how genuine co-design has consistently succeeded in Indigenous and non-Indigenous health research, leading to consumer-led health initiatives and programs that support health and well-being outcomes.^9^ These programs demonstrate what genuine co-design can look like in practice: community prioritisation, methods that include diverse worldviews and local governance, investment in local community capacity, and efforts to sustain program delivery beyond the research project's duration.^6^ They contrast with more superficial approaches, in which consumers are consulted but not placed at the center of the research process.

Recent reviews of co-design in public health highlight both the rising momentum and notable gaps in how co-design is practiced and reported.^1^^,^^9^^,^^10^ Vargas et al.^1^ examined co-designed concepts, processes, models, and frameworks in public health research, noting a surge in publications in 2023, with most studies originating from Oceania, particularly Australia. Although over 64% of the studies utilised named frameworks, nearly half did not explicitly define co-design; participant roles varied from “informants” to genuine “co-designers”; many studies did not include prototype development or testing.^1^ These findings reflect anecdotal evidence that co-design is frequently used but often enacted in markedly different ways, ranging from consultation to end-user-led processes, and reported inconsistently, which hampers learning and scaling up.^9^^,^^10^ These gaps, in design or reporting, obscure critical insights into what works, how, why, and in which cultural contexts.

Unlike established reporting frameworks such as GRIPP2 for consumer involvement, GUIDE (Guidance for Reporting Involvement of Patients and the Public), or CONSORT-Equity extensions, no consensus guidelines yet exist for reporting co-design processes in health research. Without proper reporting on key processes, methods, and outcomes, even a well-executed co-design project could be seen merely as “consultation”.^10^ Transparent and detailed reporting of the co-design process is just as vital as its actual conduct for three main reasons.

1. **Relational accountability:** Detailed reporting recognises the leadership and efforts of consumers and other co-design partners, acknowledges their ownership and governance, and fosters trust among research teams and communities.
2. **Transferability and scale-up:** When the co-design stages, roles, governance, cultural protocols, data governance, and outcomes are clearly outlined, other groups can assess the co-design process, its applicability to their context and can adapt and learn rather than reinvent.
3. **Knowledge sovereignty and empowerment:** Reporting how community decisions on the co-design process and outcomes were made, and how data and findings are returned to communities, aligns with Indigenous data-sovereignty principles and best practice guidelines. This ensures that research knowledge remains under community control and accessible for the benefit of the community.

Enhancing co-design research practices and their reporting in the Western Pacific region (and globally) is both an ethical obligation and a strategic opportunity. It ensures that consumers are not just recipients but also creators and owners of research and service systems that impact their lives. In co-designed projects with Indigenous communities, science becomes more inclusive, compassionate, and effective when Indigenous epistemologies and relational frameworks are central. Investing in transparent and culturally grounded co-design conduct and reporting can build trust, support policies shaped by consumer voices, and create a more collaborative research landscape to maximise the impact of co-design.

## Contributors

Yaqoot Fatima and Bushra Nasir conceptualised the commentary. All authors reviewed and edited it.

## Declaration of interests

We declare no conflicts of interest.
